# Usefulness of local therapy for distant metastases of differentiated thyroid cancer

**DOI:** 10.1530/ETJ-24-0237

**Published:** 2025-06-11

**Authors:** Sueyoshi Moritani, Masao Takenobu, Masakazu Yasunaga, Taihei Fujii, Hiroya Kitano

**Affiliations:** Omi Medical Center, Center for Otolaryngology, Head and Neck Thyroid Surgery, Kusatsu, Shiga, Japan

**Keywords:** local therapy, thyroid cancer metastases, quality of life, bone-modifying agents

## Abstract

**Objective:**

Treatment for distant metastases (DM) of differentiated thyroid cancer (DTC) aims to improve the prognosis and quality of life (QOL). Radioactive iodine and molecular targeted therapies are the primary systemic treatments for DM. Combining these with local treatments, such as surgery and radiotherapy targeting metastatic sites, may provide additional benefits to systemic therapy.

**Methods:**

This study reviewed the additional effects of local therapies on common metastatic sites of DTC, such as pulmonary or bone metastases (BM), the efficacy of bone-modifying agents (BMAs) for BM, and the therapeutic effects of local treatments for less common brain metastases.

**Results:**

Although based on retrospective studies with no definitive conclusions on the effectiveness of each treatment, local therapy for DM in DTC patients has been shown to enhance prognosis and QOL in several studies. Considering the results of clinical trials for metastatic tumors, local therapy for one or a few pulmonary or BM in DTC should be considered, if expected to improve prognosis and QOL. Surgical intervention is recommended for spinal metastases presenting with spinal compression symptoms or for long BM in extremities at risk of pathological or impending fractures. BMAs are also recommended to reduce the risk of skeletal-related events. Surgery, stereotactic radiotherapy, and whole-brain radiotherapy are suggested for brain metastases to improve the prognosis and QOL.

**Conclusion:**

Incorporating local therapy alongside systemic treatment can enhance the prognosis and QOL and should be considered a viable treatment option.

## Introduction

Differentiated thyroid cancer (DTC), which accounts for most thyroid cancers, has an excellent prognosis, with a 10-year survival rate of over 90%. However, local recurrence has been reported in 5–20% of cases and distant metastasis (DM) in 10–15%. The most common site of DM is the lung (49%), followed by the bone (25%). Simultaneous pulmonary and bone metastases (BM) occur in approximately 15% of cases. The most common sites of BM include the spine (34.6%), pelvis (25.5%), sternum and ribs (18.3%), and extremity bones (15.6%), often in areas with abundant blood flow. Moreover, BM in DTC typically exhibits an osteolytic pattern, and half of the metastases are solitary (1, 2). DM is more prevalent in follicular thyroid carcinoma (7–28%) than in papillary thyroid carcinoma, where it ranges from 1.4 to 7%, depending on the histological type (1, 3).

The first-line treatment for DM is radioactive iodine (RAI) therapy, provided no emergency treatment is required. For RAI-refractory cases, molecular targeted therapy is initiated based on the degree of tumor progression. A study examining the prognosis of 1,991 patients with distant metastatic DTC identified from the surveillance, epidemiology, and end results-13 cancer registry program, divided into three time periods (1992–1998, 1999–2008, and 2009–2018), reported 10-year disease-specific survival rates of 50.2, 47.3, and 52.4%, respectively (*P* = 0.48). No significant difference was reported in prognosis across these three periods. However, the prognosis for DTC patients with DM is expected to improve with the introduction of selective tyrosine kinase inhibitors (4).

Treatment for DM aims to improve prognosis and quality of life (QOL). RAI and molecular targeted therapies are the primary systemic treatments for DM. When combined with local treatments, such as surgery and radiotherapy targeting metastatic sites, these therapies may offer additional benefits to systemic therapy. This study reviews the additional effects of local therapies on common metastatic sites of DTC, such as pulmonary or BM, the efficacy of bone-modifying agents (BMAs) for BM, and the therapeutic effects of local treatments for less frequent brain metastases.

## Systemic therapy for DTC with DM: RAI therapy and molecular targeted therapy

### RAI therapy

Systemic treatment options for DTC with DM include RAI and molecular targeted therapy. The first treatment to consider is RAI therapy, with the decision based on factors such as lesion location, number, size, and progression. It has become evident that the success of RAI therapy is significantly influenced by histologically aggressive DTC subtypes, such as the tall cell variant and progressive genetic profiles, including *BRAF* and *TERT* promoter mutations. The lungs are the most promising metastatic site for RAI therapy, particularly when I-131 uptake is observed in micronodular lung metastases, which often lead to favorable treatment outcomes. However, the effectiveness of RAI therapy decreases in patients over 40 years of age or those with large nodular metastases (5, 6). BM are another common site for DM, following lung involvement. The median 5- and 10-year overall survival rates from BM diagnosis have been reported as 56.5–63.5% and 15.3–36.7%, respectively (7, 8, 9, 10). Even when RAI uptake is observed in BM, the rate of complete response is not necessarily high. Brain metastases in DTC are relatively rare, occurring in 0.15–1.5% of cases. RAI should be avoided for brain metastases as few brain metastatic lesions take up RAI, and when uptake does occur, it may lead to serious complications such as cerebral edema and cerebral hemorrhage (11).

### Molecular targeted therapy

Molecular targeted therapy includes multikinase inhibitors (MKIs) and selective kinase inhibitors. For unresectable recurrent or metastatic RAI-refractory DTC (RAIR-DTC), two MKIs are available: sorafenib and lenvatinib. These drugs primarily target vascular endothelial growth factor receptors, inhibiting tumor angiogenesis and thereby slowing disease progression. The efficacy of these two drugs was demonstrated in phase III randomized-controlled trials (the DECISION and SELECT trials) targeting unresectable recurrence or metastatic RAIR-DTC (12, 13). Even in cases of RAIR-DTC, disease progression can be slow, and immediate initiation of MKI therapy may not always be advisable due to the high rate of adverse events and the need for lifelong therapy or until disease progression occurs. Given that MKIs can cause various side effects that may impair patients’ QOL, it is recommended to consider initiating MKI therapy only if disease progression occurs within 6–12 months. Selective tyrosine kinase inhibitors have recently expanded the options for patients with unresectable or metastatic DTC. BRAF/MEK inhibitors, such as dabrafenib/trametinib, are available for patients with *BRAF*-mutant RAIR-DTC. The RET inhibitor selpercatinib is an option for *RET* fusion-positive cases, while TRK inhibitors entrectinib and larotrectinib are approved for *NTRK* fusion-positive DTC. A phase II study (*n* = 53) comparing dabrafenib with dabrafenib plus trametinib in *BRAF*-mutated RAIR-DTC reported a response rate of 48% (RECIST 1.1: 30%), with a median progression-free survival of 15.1 months and overall survival of 47.5 months for combination therapy (14). In the LIBRETTO-001 trial, selpercatinib demonstrated a 79% response rate and a 1-year progression-free survival of 64% in *RET* fusion-positive thyroid cancer patients (*n* = 19) (15). For *NTRK* fusion-positive thyroid cancer, entrectinib showed a 54% response rate and a median progression-free survival of 19.9 months (*n* = 13) (16), while larotrectinib showed an 86% response rate and a 1-year progression-free survival of 100% in evaluable DTC patients (*n* = 21) (17). These selective tyrosine kinase inhibitors have demonstrated high efficacy, safety, and tolerability in single-arm trials and integrated analyses of these trials. Therefore, selective tyrosine kinase inhibitors are proposed as a first-line molecular targeted therapy for RAIR-DTC with driver gene mutations or fusion-positive profiles. Treatment options for unresectable or metastatic DTC have become increasingly complex, even in the case of molecular targeted therapy, particularly regarding the timing of initiation, selection of agents, and management of their side effects. In addition to molecular targeted therapy, local treatment options such as surgery or radiation therapy for distant metastatic lesions require input from a multidisciplinary team, including surgeons, oncologists, and nuclear medicine specialists, to determine appropriate treatment strategies and effectively manage side effects.

## Local therapy for BM and the role of BMAs

### Local therapy for one or a few BM

Surgical treatment may improve the prognosis and QOL if the lesion is resectable, particularly for one or a few BM, including those in the spine and long bones of the extremities (5, 6). In cases of BM without pathological fractures or spinal cord compression symptoms, radiation therapy can be expected to relieve or eliminate symptoms. A meta-analysis of randomized-controlled trials assessing the efficacy of radiation therapy for metastatic bone tumors revealed that radiation relieved pain in 61–62% of patients in an intention-to-treat analysis (72–75% in assessable patients) and eliminated pain in 23–24% (28–29% in assessable patients) (18). These findings demonstrate the effectiveness of radiation therapy in managing pain associated with symptomatic BM.

All studies on local therapy for BM in DTC using surgery or radiotherapy are retrospective and predominantly from single centers. The added effect of local therapy in conjunction with RAI treatment remains unclear (19, 20, 21, 22, 23, 24, 25, 26, 27, 28, 29). In a study of 93 RAI-avid patients with BM from DTC, Talaat *et al.* (19) reported that the disease control rate was higher with the combination of radiotherapy with RAI than with RAI therapy only (complete response rate, partial response plus stable disease rate, and progression disease rate; 9.0, 78.3, 12.7% vs 7.9, 56.8, and 23.7%, respectively, *P* = 0.03). Similarly, in a study of 145 RAI-avid DTC patients with BM, Jannin *et al.* (20) reported that RAI therapy combined with BM focal treatment, such as BM surgery, radiotherapy, thermal ablation, and cementoplasty, increased the complete BM response rate from 18 to 31.7% compared with that noted with RAI therapy alone. Wu *et al.* (21) reported an improvement in the median overall survival from 3.9 to 7.7 years when RAI therapy was combined with BM focal treatment compared to RAI therapy alone in a study of 77 patients with BM from DTC (*P* = 0.03). However, in a nationwide survey of 143 patients with BM from DTC, which reflected real-life management rather than a systematic therapeutic approach to BM, Mazziotti *et al.* (22) reported that the overall mortality significantly decreased by RAI therapy (HR 0.10; *P* = 0.02), whereas no significant effects were induced by BMAs (*P* = 0.36), external radiotherapy (*P* = 0.54), and surgery (*P* = 0.43) for BM.

Regarding the additive effect of BM surgery on RAI therapy, Qiu *et al.* (23) identified solitary BM, bone-only metastasis, and the combination of RAI therapy with BM surgery as independent factors associated with improved prognosis. Similarly, Orita *et al.* (24) and Fragnaud *et al.* (25) reported a better prognosis in patients who underwent BM surgery than in those who did not, suggesting that surgical resection should be considered, especially for solitary BM. In a study of 109 patients with BM from DTC, including 53 patients with synchronous or metachronous metastases in other organs, Bernier *et al.* (26) found that complete resection of BM contributed to improved survival (Table 1). These findings underscore the potential benefit of local therapy, particularly for patients with one or a few BM, in preserving QOL.

**Table 1:** Local therapy for single or a few BM of thyroid cancer.

Author	Patients with BM	Intervention: *n* (%)	Comparison: *n*	Outcomes
Talaat *et al.* (19)	93 RAI-avid	RAI + RT: 55	RAI only: 38	RAI + RT resulted in a higher disease control rate than RAI only
Jannin *et al.* (20)	145 RAI-avid	RAI + BMFT		46 (31.7%) patients achieved a complete BM response following RAI treatment, either alone in 32 (18%) patients or in association with BMFT modalities in 14
Wu *et al.* (21)	77 DTC	RAI + BMFT: 54 (70%)	RAI only: 23 (30%)	Improvement in the median overall survival from 3.9 to 7.7 years when RAI therapy was combined with BMFT compared to RAI therapy alone (*P* = 0.03)
Mazziotti *et al.* (22)	143 DTC	RAI: 131 (91.6%), BMFT – surgery or EBRT: 68 (57.6%), BMAs: 32 (22.4%)		The overall mortality was significantly decreased by RAI therapy (HR: 0.10; *P* = 0.02), whereas no significant effects were induced by bone-modifying agents (*P* = 0.36), external RT (*P* = 0.54), and surgery (*P* = 0.43) of BM
Qiu *et al.* (23)	106 DTC	Surgical treatment of BM before RAI: 26	RAI only: 80	The 10-year survival was significantly longer in the group with a combined BM surgery (69.7 vs 45.1%, *P* = 0.039, log-rank test)
Orita *et al.* (24)	52 DTC	BM surgery: 11, RAI: 26	No BM surgery: 41, no RAI: 26	5-year DSS, BM surgery performed 60% vs not performed 37% (*P* = 0.050, log-rank test), RAI performed 59% vs not performed 23% (*P* = 0.0028, log-rank test). The 5-year DSS was significantly longer in the group with BM surgery
Fragnaud *et al.* (25)	40 thyroid cancer	*en block* resection for BM		Survival for patients with single bone metastasis at 5 and 10 years was 82.3 and 82.3%, respectively, compared with 0 and 0% (log-rank, *P* = 0.022) for patients with multiple BM.
Bernier *et al.* (26)	109 DTC	RAI treatments + BMFT (BM surgery: 84 (77%), external RT: 39 (36%), SAE: 34 (31%)		Complete BM surgery was significantly associated with improved survival (*P* < 0.02)

RAI, radioactive iodine; BM, bone metastases; DTC, differentiated thyroid cancer; EBRT, external beam radiotherapy; BMAs, bone-modifying agents; HR, hazard ratio; RT, radiotherapy; BMFT, bone metastases focal treatment; SAE, selective arterial embolization.

### Surgical treatment of spinal metastases (SM) with spinal compression symptoms or long BM in extremities at risk of pathologic or impending fracture

Spinal cord compression caused by BM can result in complete paralysis within hours, making early treatment essential to prevent irreversible damage. Although evidence on surgical therapy for SM is limited, Patchell *et al.* (30) demonstrated that surgery followed by radiotherapy significantly improves outcomes compared to radiotherapy alone for patients with spinal cord compression due to metastatic bone tumors. In the surgery group, 84% of patients regained the ability to walk (OR: 6.2, 95% CI: 2.0–19.8, *P* = 0.001) compared to the 57% in the radiotherapy-only group. In addition, the ability to walk lasted longer in the surgery group (median: 122 vs 13 days, *P* = 0.003). For long BM, progression can lead to severe pain and pathological fractures, thereby impairing limb function. A multicenter study by Nooh *et al.* (31) reported significant improvements in limb function and pain as early as 2 weeks post-surgery, with continued improvement up to 1 year. These findings highlight the efficacy of surgical intervention in improving the QOL for patients with metastatic long bone disease.

In a retrospective analysis of 202 patients with thyroid cancer SM, 57% presented with symptoms of spinal cord compression, including spinal cord compression in 45% and root compression in 12%. Back pain was the initial symptom in 28% of patients, while the remaining 15% were asymptomatic. Among the 183 cases with treatment data for SM, surgical therapy was selected for 67%, external irradiation for 47.5%, and arterial embolization for 39%. Due to the variability in SM progression and treatment options, there is limited literature comparing the effectiveness of treatments for thyroid cancer-related SM (3).

In a retrospective study of 50 DTC patients with cervical spine metastases, Yin *et al.* (32) reported that the 5-year overall survival after diagnosis of cervical spine metastases was better in the surgery group than in the non-surgery group (44.7 vs 11.1%, *P* = 0.01) and surgical intervention was significantly associated with improved survival (HR = 0.37, 95% CI: 0.14–0.98, *P* = 0.04, multivariate analysis). Jiang *et al.* (7) reported that surgery combined with conservative treatment or total spondylectomy for DTC-SM did not affect postoperative recurrence or survival. However, curettage and stabilization could effectively relieve pain and improve patients’ QOL and neurological status. Conversely, Demura *et al.* (33) or Kato *et al.* (34) reported that surgical technique influenced local tumor recurrence and prognosis for thyroid BM. Patients undergoing complete excision had better survival than those with incomplete excision (5-year survival: 84 vs 50%; 10-year survival: 52 vs 8%; *P* < 0.01). They concluded that complete surgical resection of thyroid SM, when feasible, can help maintain performance status and improve survival.

Conversely, some studies have suggested that surgery may not contribute to improved prognosis. Planty-Bonjour *et al.* (2) found that among 51 patients treated for BM, ten underwent posterior decompression laminectomy. Univariate analysis indicated that good ECOG-PS (status 0 and 1), ambulatory status, and no epidural involvement (*P* = 0.01) were associated with improved survival but not with spine surgery (*P* = 0.937). In the multivariate analysis, better performance status was associated with improved survival. Specifically, ECOG-PS of 0 (HR: 0.3, 95% CI: 0.1–0.941; *P* < 0.0001) and 1 (HR: 0.8, 95% CI: 0.04–2.124; *P* = 0.001) were independent predictors of better prognosis. In addition, patients with ambulatory neurological status classified as Frankel E also showed better survival outcomes (HR: 0.262, 95% CI: 0.048–1.443; *P* = 0.02). Zhang *et al.* (35) examined prognostic factors in 52 patients with spinal cord compression due to thyroid cancer SM who underwent surgical treatment. Of these, eight patients had en-bloc tumor resection and the remaining 44 underwent curettage. The 5-year survival rate for all patients was 78.8%. Favorable prognostic factors included being ≤50 years old, having a single segment involved, and having follicular thyroid cancer. The type of treatment, including surgery, did not show statistical significance. In a study of 43 patients undergoing spinal surgery for BM, Sellin *et al.* (36) reported that progressive systemic disease at the time of spine surgery and postoperative complications were associated with worse overall survival (HR: 8.98, 95% CI: 3.46–23.30, *P* < 0.001; and HR: 2.86, 95% CI: 1.30–6.31, *P* = 0.009, respectively). The preoperative neurological deficit was also significantly associated with worse overall survival (HR: 3.01, 95% CI: 1.34–6.79, *P* = 0.008). However, spinal surgery was not found to be a prognostic factor. Good ECOG-PS and minimal or no neurological symptoms were favorable prognostic factors for patients with BM, although metastatic site, number of metastases, and disease progression varied among individuals (Table 2).

**Table 2:** Surgical treatment of SM of thyroid cancer.

Study	Patients	Intervention (*n*)	Comparison (*n*)	Outcomes
Yin *et al.* (32)	50 DTC with cervical SM (PTC: 34, FTC: 8, HCC: 8)	Surgical treatment (16)	Nonsurgical treatment (34)	5-year overall survival after diagnosis of cervical SM was better in the surgery group than in the nonsurgery group (44.7 vs 11.1%, *P* = 0.01). Surgical intervention was significantly associated with improved survival (HR = 0.37, 95% CI: 0.14–0.98, *P* = 0.04, multivariate analysis)
Jiang *et al.* (7)	21 DTC with SM (PTC: 4, FTC: 17)	Total spondylectomy intralesionally or piecemeal excision (3)	Curettage + stabilization (18)	There was no difference in postoperative recurrence or survival by surgical technique. Curettage and stabilization can effectively relieve the pain and improve the QOL.
Demura *et al.* (33)	24 TC with SM (PTC: 8, FTC: 15, MTC: 1)	TES (10)	Piecemeal excision or eggshell curettage (14)	5-year survival rates for patients after TES and debulking surgery were 90 and 63%, respectively, with no difference. However, local recurrence was significantly lower with TES.
Kato *et al.* (34)	32 TC with SM (PTC: 10, FTC: 21, MTC: 1)	Complete resection (20)	Incomplete resection (12)	Patients undergoing complete excision survived longer than those undergoing incomplete excision (5-year survival: 84 vs 50%; 10-year survival: 52 vs 8%; *P* < 0.01)
Planty-Bonjour *et al.* (2)	51 TC with SM (PTC: 22, FTC: 24, MTC: 5)	Posterior decompressive laminectomy (10)	Non-surgical treatment (41)	Favorable ECOG-PS and ambulatory neurological status (Frankel E) were independent predictors of better survival. Surgical treatment did not affect the prognosis
Zhang *et al.* (35)	52 TC with spinal cord compression due to SM (PTC: 7, FTC: 43, MTC: 2)	*En-block* tumor resection (8), curettage (44)		Age ≤50 years, single segment involved, and FTC are favorable prognostic factors for TC patients with SM.
Sellin *et al.* (36)	43 TC with SM (PTC: 9, FTC: 20, MTC: 6, HCC: 6, PDTC: 2)	Surgical treatment (43) with several preoperative treatments		Progressive systemic disease, postoperative complications, and preoperative neurological deficits were significantly associated with worse overall survival, while preoperative spinal embolization was associated with improved overall survival

TC, thyroid cancer; DTC, differentiated thyroid cancer; PTC, papillary thyroid cancer; FTC, follicular thyroid cancer; HCC, Hurther cell carcinoma; MTC, medullary thyroid cancer; PDTC, poorly differentiated thyroid cancer; QOL, quality of life; SM, spinal metastases; TES, total en-block spondylectomy; EOCG-PS, Eastern Cooperative Oncology Group-performance status.

Based on the above, due to the diversity of treatment options and limited literature comparing their effectiveness, no definitive conclusion can be drawn regarding whether surgery improves prognosis. However, surgery may improve prognosis and QOL, particularly in cases where complete tumor resection is achieved. In addition to surgical therapy, factors such as performance status, systemic disease progression, and neurological symptoms often play a more critical role in prognosis. Given the variability in patient conditions, personalized treatment plans are essential.

### Role of BMAs

Skeletal-related events (SREs) associated with the progression of BM significantly impact patients’ daily lives, QOL, and prognosis. Preventing SREs is clinically essential. In a study of 245 patients with BM from DTC, 78% experienced at least one SRE. The interval from BM diagnosis to the first SRE was 5 months, with 65% experiencing a second SRE. The median time between the first and second SREs was 10.7 months, similar to the intervals observed in metastatic breast and prostate cancer (37). The mortality rate was significantly higher in patients with BM who developed SREs – 68% after a single SRE and 79% after multiple SREs – than the 42% in those who did not (*P* = 0.0001) (29). Given the high incidence of SREs and poor prognosis in patients with BM from thyroid cancer, BMAs should be initiated early after diagnosis to delay the onset of SRE.

To the best of our knowledge, no randomized study to date has examined the effect of BMAs on thyroid cancer. In a retrospective study of 50 DTC patients with BM, Orita *et al.* (38) compared the incidence of SREs with and without zoledronic acid. They found that the frequency of SREs was significantly lower in the treatment group than in the non-treated group (14 vs 50%, *P* = 0.007). They also reported that the use of zoledronic acid significantly delayed the onset of the first SRE (*P* = 0.04). Furthermore, in a prospective, single-arm study of 19 DTC patients with BM treated with zoledronic acid, eight patients developed SREs, but only one patient had metastatic spinal cord compression. In 68% of patients, pain improved or remained unchanged, suggesting that zoledronic acid may play a role in the multidisciplinary treatment of patients with BM (39).

In phase III, a double-blind, randomized trial of skeletal metastases in patients with lung cancer and other solid tumors, compared to placebo, zoledronic acid significantly delayed the time to the first SRE, with a median time of 230 versus 163 days for the placebo (*P* = 0.023). This is a crucial outcome for a population with a poor prognosis. In addition, a multiple-event analysis showed that zoledronic acid significantly reduced the risk of SREs, with a hazard ratio of 0.732 (*P* = 0.017) (40). A randomized, double-blind study comparing denosumab and zoledronic acid in the treatment of BM in patients with advanced cancer (excluding breast and prostate cancer) or multiple myeloma demonstrated no difference in overall survival or disease progression. However, it showed the non-inferiority of denosumab compared with zoledronic acid in delaying the time to the first on-study SRE (HR: 0.84; 95% CI: 0.71–0.98, *P* = 0.0007) (41). Moreover, in a subanalysis of this study, denosumab significantly delayed the time to first SRE or hypercalcemia of malignancy (Kaplan–Meier estimate, median 19.0 months for denosumab vs 14.4 months for zoledronic acid; HR: 0.83, 95% CI: 0.71–0.97, *P* = 0.022). Denosumab also significantly reduced the risk of radiation to the bone by 22% (HR: 0.78, 95% CI: 0.63–0.97, *P* = 0.026) (42). Adverse events were generally similar, but hypocalcemia and osteonecrosis of the jaw were more commonly reported with denosumab, while nephrotoxicity and acute phase reactions such as pyrexia and influenza-like symptoms were more common with zoledronic acid (43, 44, 45). Although there are no prospective studies of BMAs for BM of thyroid cancer, the results of these randomized trials suggest that BMAs should be aggressively applied to BM in thyroid cancer.

## Local therapy for one or a few pulmonary metastases (PM)

All reports on local therapy of DTC PM with surgery or radiotherapy are based on retrospective studies focusing on prognosis. Regarding the impact of pulmonary resection on PM, Porterfield *et al.* (46) reported a 64% 5-year survival rate after thoracic metastasectomy in 31 PTC patients, with 17% experiencing postoperative complications. They concluded that thoracic metastasectomy should be offered to patients with thoracic metastasis who can tolerate surgical resection and for whom either complete resection is achievable or resection of progressing dominant lesions is possible. In a study of PM for 43 patients of RAIR-DTC, Moneke *et al.* (47) reported that the prognosis of patients who underwent R0 resection was significantly better than that of the patients who underwent incomplete resection (*P* = 0.013).

Expert consensus on local therapy for metastatic pulmonary tumors from The Society of Thoracic Surgeons Work Force of Evidence-Based Surgery states that: i) pulmonary metastasectomy should be considered within a multidisciplinary team and carefully individualized for patients with cancer and pulmonary oligometastases; ii) pulmonary metastasectomy should be considered with preference given to minimally invasive surgery; iii) although the absolute number of PM is not a direct contraindication, candidates for pulmonary metastasectomy are best suited to patients with three or fewer PM; and iv) stereotactic radiotherapy is a reasonable option for patients with pulmonary oligometastases, particularly for those at high risk for resection or those who refuse resection (48). Based on these considerations, local therapy for PM in DTC should be tailored to each patient’s condition. If complete removal or surgical resection of progressive invasive lesions is possible, surgery is recommended as the primary option, with the potential use of minimally invasive techniques and radiotherapy when appropriate.

## Local therapy for brain metastases

The frequency of brain metastases in DTC is low, ranging from 0.15 to 1.5%, and approximately 60% of patients with brain metastases have concurrent PM (49, 50). The treatment option should consider the presence and control of the primary tumor and other metastatic lesions, the patient’s general condition and age, and the status of brain metastases. All studies on the treatment of brain metastases from thyroid cancer are retrospective, mostly from single centers and with a small number of cases. According to 12 studies collected (49, 50, 51, 52, 53, 54, 55, 56, 57, 58, 59, 60), the 1- and 2-year overall survival rates ranged from 28 to 68.2% and 30.2–45.5%, respectively.

Treatment options for brain metastases from DTC include surgery, stereotactic radiotherapy, whole-brain radiotherapy, chemotherapy, molecular targeted therapy, and RAI therapy, either alone or in combination. Surgery and stereotactic radiotherapy are the most common treatment choices, with patients selected for these options showing better prognoses compared to those who receive other treatment options. Stereotactic radiotherapy, in particular, has been increasingly chosen in recent years (59). As there is no evidence-based treatment specifically for brain metastases from DTC, treatment options should align with those recommended for metastatic brain tumors in adults. These include surgery, stereotactic radiotherapy, and whole-brain radiotherapy. Prognostic indicators such as Recursive Partitioning Analysis (RPA) and Graded Prognostic Assessment (GPA) take into account factors such as Karnofsky Performance Status (KPS) score, age, control of the primary tumor, and presence of extracranial metastases (61). The patient’s overall condition and age at the time of treatment are critical factors in determining the appropriate approach for brain metastases.

Tumor resection is recommended for brain metastases when there are four or fewer lesions, located in resectable regions, and with a diameter of 3 cm or more. For lesions smaller than 3 cm, stereotactic radiotherapy or surgery is recommended. Whole-brain radiotherapy is advised for patients with five or more brain metastases. However, for up to ten lesions with a total volume of 15 mL or less, stereotactic radiotherapy with careful follow-up and salvage therapy is also an option (62, 63, 64, 65). While whole-brain radiotherapy is the standard treatment for multiple brain metastases, cognitive decline has been reported 3–4 months after irradiation. In addition, adding whole-brain radiotherapy after stereotactic radiotherapy or surgery can reduce local recurrence and the appearance of new lesions but does not improve survival (66, 67, 68, 69, 70). Given that brain metastases in DTC are rare and there is no evidence-based treatment specifically for them, individualized treatment plans are essential. While surgery and stereotactic radiotherapy are preferred for limited metastases, whole-brain radiotherapy remains an option for multiple lesions despite the associated cognitive risks.

## Conclusions or future perspectives

The study findings indicate that although limited to retrospective studies and lacking definitive conclusions on the effectiveness of each treatment, local therapy for DM in patients with DTC has been shown to improve prognosis and QOL in many cases. Combining local therapy with systemic treatment is expected to further enhance QOL and prognosis and should be considered a viable treatment option.

## Declaration of interest

The authors declare that there is no conflict of interest that could be perceived as prejudicing the impartiality of the work reported.

## Funding

This work did not receive any specific grant from any funding agency in the public, commercial, or not-for-profit sectors.

## Ethics approval

The Ethical Committee of Omi Medical Center approved this study (Research Project No. 2024-0042).
